# Experimental and Numerical Study on Interface Bond Strength and Anchorage Performance of Steel Bars within Prefabricated Concrete

**DOI:** 10.3390/ma14133713

**Published:** 2021-07-02

**Authors:** Zhijian Hu, Yasir Ibrahim Shah, Pengfei Yao

**Affiliations:** 1 Department of Road and Bridge Engineering, School of Transportation, Wuhan University of Technology, Wuhan 430070, China; hzj@whut.edu.cn; 2 Central and Southern China Municipal Engineering Design & Research Institute Co., Ltd., Wuhan 430010, China; yaopf@citic.com

**Keywords:** bond strength, prefabricated concrete structure, anchorage performance, mechanical bond strength

## Abstract

This study investigates the interface bond strength and anchorage performance of steel bars within prefabricated concrete. Twenty-two specimens were designed and manufactured to study the interface bond behavior of deformed and plain steel bars under a larger cover thickness. Diameter of steel bars, strength grade of concrete, and anchorage length were considered influential factors. The finite element method (ABAQUS) was used for the validation of experimental results. The interface bond’s failure mechanism and the anchorage length in the prefabricated concrete under different concrete strength levels were explored and compared to national and international codes. A suitable value of the basic anchoring length for the prefabricated structure was recommended. The results show that the interface bond strength of prefabricated bridge members is directly proportional to the strength grade of the concrete, inversely proportional to the reinforcement diameter, and less related to anchorage length. The effect of the cover thickness of the surrounding concrete is negligible. Conversely, the bearing capacity of prefabricated bridge members depends on the strength of the concrete, the diameter of the steel bar, and the anchorage length. Furthermore, it is concluded that the mechanical bond strength accounts for 88% of the bond strength within prefabricated concrete.

## 1. Introduction

In recent years, prefabricated reinforced concrete structures are widely used to construct commercial buildings, temporary safety protection structures, and large and medium-size bridges. The prefabricated structure has the advantages of a short construction periods, industrialized production, high dimensional accuracy, and less environmental pollution. The sufficient bond strength and anchorage performance of steel bars within prefabricated concrete are the key to ensure the service performance of the structure [1,2,3].

At present, the design of the anchorage length of the connecting reinforcement within the prefabricated concrete structure is usually considered according to the relevant provisions of the cast-in-place structure. However, due to the different characteristics of the prefabricated connection, the thickness of the protective layer of the connecting reinforcement is generally more than 50 mm larger than that of the cast-in-place structure and so the influence of the thickness of the protective layer can be ignored in the calculation of the anchorage length and interface bond strength. Presently, the existing relevant specifications of the assembled concrete structure still utilizes the relevant provisions of the cast-in-place structure for the anchorage length of the connecting steel bars, which is not suitable for calculating the bond strength and anchorage performance of the prefabricated assembled bridge with large protective layer thickness.

There are only a few publications for the case of prefabricated concrete that have been published; this is the motivation for conducting the presented research. For the general case of a bond between reinforcement and concrete, however, much work has been conducted and reported in publications; the most relevant is mentioned here and shortly discussed. Steel bars have a significant effect on the mechanical properties and bond strength of concrete [4]. Li et al. established the formula of the ultimate bond strength by an experimental study on bond anchorage performance of 1860-grade high-strength prestressed steel strands and lightweight aggregate concrete [5].

The experimental study analyzes the compressive bond anchorage properties of 500 MPa steel bars in concrete. Five influence factors, including concrete strength, the steel bar’s diameter, concrete cover, embedment length, and transverse reinforcement, were considered. The result shows that the influence of the surrounded concrete cover thickness on compressive bond strength is more than the steel bar’s diameter [6]. Saeed et al. concluded from an experimental study that the anchor strength and stiffnesses are directly proportional to the bond length; the cross-sectional area ratio of Carbon Fibre-Reinforced Polymer (CFRP) rods to anchor borehole affects the stiffness and bonding capacity of the anchor [7]. Dang et al. proposed the standard test to investigate the bond performance of 18 mm prestressing strands used in precast/prestressed concrete applications. The pull-out resistance of steel can be improved by controlling the crack growth inside the concrete [8]. Hayashi et al. used the three-dimensional discrete model to analyze reinforced concrete (RC) anchorage performance. Results indicate that concrete strength is reduced if reinforcement spacing between the column and the embedded is very close because of a non-homogeneous behavior of concrete anchorage performance in a multidirectional arrangement of reinforcement bars [9].

Bond performance between concrete and steel bars was examined under corrosion level and temperature and it was found, from the study, that bond strength was influenced by temperature and corrosion [10]. John et al. revised how the anchorage’s contribution was calculated and recognized the contribution of end bearing to laps and anchorages of compression bars. Bond influences the width and spacing of transverse cracks, tension stiffening, and flexural curvature. At the ultimate limit state, the bond is responsible for the strength of end anchorages and lapped joints of reinforcement and influences the rotation capacity of plastic hinge regions [11]. The hysteretic behavior of the anchorage slip is examined in reinforced concrete structures. Reinforced concrete columns subjected to axial compression and inelastic lateral deformation reversals develop significant rotations due to anchorage slip [12].

Anchorage and pull-out behavior depend on the geometry of steel fibers and is also related to the characteristics of the matrix [13]. The form of wet connection is to weld, lap, or mechanically connect the reserved connecting steel bar or connecting rod at the connection part, anchor the steel bar through post cast concrete or other grouting materials, and to connect the different prefabricated components. The dry connection is to embed the steel connecting parts in the prefabricated concrete components and then to connect them into one through bolt connection or welding a holistic approach [14]. The failure mode of reinforced concrete central pull-out specimen and the whole failure process of the reinforced concrete bond interface is divided into two stages: the elastic stage without crack and the working stage with crack. Based on the experimental results, the corresponding calculation methods of bond interface energy in different stages are obtained. The process of bond failure of reinforced concrete was analyzed from the perspective of energy [15].

Much numerical research has been carried out on the stochastic character of concrete [16,17,18,19,20,21]. Concrete is a primary construction material composed of cement and aggregates and the geometry and distribution of aggregates significantly affect the interface bond performance of the concrete structure. Jonak et al. used a contact interface between concrete and undercut anchor by using the finite element method (ABAQUS) and studied the cone failure occurring in the pull-out test. The result shows that the break-out angle of the undercut anchor was considerably less than the concrete capacity design method [18,19]. Ombres et al. conducted direct single-lap shear tests on 20 specimens in order to study the bond behavior of steel reinforce grout to concrete joints. Experimental results were compared with finite element simulation and spectated to be in good agreement [21]. Funari et al. proposed a moving mesh numerical model using interface elements to calculate debonding mechanisms, crack opening, and cracks propagation in fiber-reinforced polymer (FRP) concrete beams [22].

It is concluded from the literature review that research on the bond strength and anchorage performance of reinforcement-concrete used in prefabricated bridges is still limited in number. There are still some differences in the design standards of the assembly type concrete and anchoring. However, due to the differences in test conditions and test design set by different scholars, the current test results are relatively discrete and the conclusions are quite different. Moreover, the influence of the failure mode on the interface bond strength calculation has not been clearly distinguished in the existing experimental studies. The complete interface failure method was used to calculate the bond strength, leading to the smaller calculated value of bond strength between steel and prefabricated concrete. A high value of the anchorage length is not suitable for the design and construction of prefabricated bridges. Existing codes for prefabricated structures stipulate the anchorage length of a post-cast straight anchor connecting steel bars of precast concrete members (JGJ 145-2004) (GB 50010-20118) [23,24]. The mechanical characteristics of reinforced concrete bonding interface under sufficient cover thickness are an essential issue in the design and construction of prefabricated bridges.

This study investigates the influential factors that affect the interface bond strength and the anchorage performance of steel bars within prefabricated concrete. Twenty-two specimens were manufactured for the pull-out test by utilizing a larger cover thickness. Based on experimental results and finite element simulation, the failure mode, ultimate load, the load-displacement curves, and the effect of the different influential factors on interface bond strength and anchorage performance of steel bars within prefabricated concrete were analyzed.

Furthermore, the bond strength calculation formulas and anchorage length for the steel bars within prefabricated concrete were fitted and derived. The national and international codes for the anchorage length and interface bond strength of steel bars within prefabricated concrete under different concrete strength levels were compared and analyzed. Recommended values of bond strength and the anchorage length of steel bars in the design of prefabricated bridges are given.

## 2. Experimental Program and Analysis of Test Results

### 2.1. Specimen Design and Fabrication

**Figure 1:**
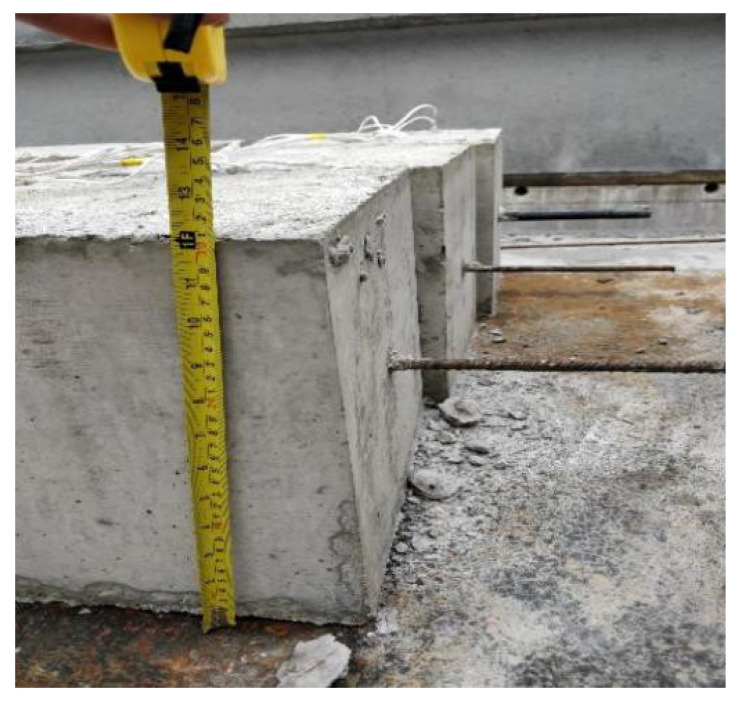
Reinforced concrete test specimens.

Twenty-two reinforced concrete specimens were designed and manufactured for pull-out tests using larger cover thicknesses are shown in Figure 1. The section size of each specimen is 300 mm × 300 mm. The concrete strength grades were C30 and C50, the anchorage lengths of reinforcement were 200 mm and 300 mm, the diameters of the ribbed steel bar were 16 mm and 20 mm, and the diameters of plain steel bars were 8 mm and 20 mm.

### 2.2. Materials Test and Properties

Hot-rolled Ribbed Bar (HRB400) [25] with the diameter of 12 mm and 16 mm was used in this study. Elastic modulus and compressive strength of C50 and C30 were calculated according to relevant specifications [26]. Three groups of standard cubes 150 mm × 150 mm × 150 mm were made for the compression test of C50 and C30 (each group has three specimens). An elastic modulus test was carried out on a prism block of the size 150 mm × 150 mm × 300 mm; three groups of specimens were designed with three test specimens in each group. The mix proportions of C30 and C50 are given in Table 1.

The tensile test was carried out according to the Chinese code [27,28] out on HRB400 with a diameter of 12 mm and 16 mm. The average value of material performance test results of concrete and steel bars are shown in Table 2.

### 2.3. Test Instruments

**Figure 2:**
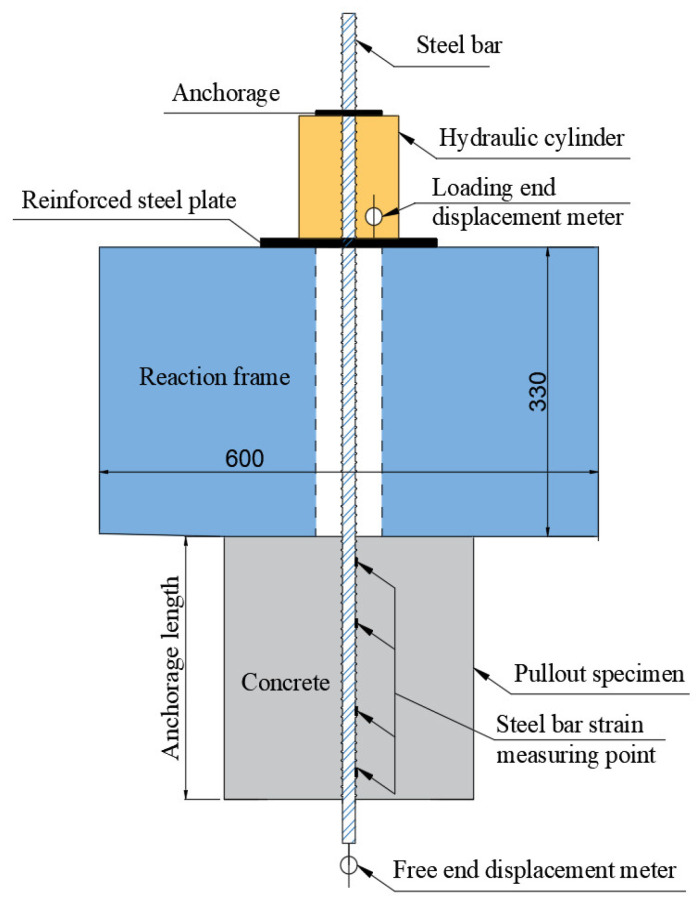
Schematic diagram of the loading device.

A bolt comprehensive parameter tester was used as a loading device. An intelligent digital pressure gauge indicates the load’s value and the loading end displacement was measured by electronic displacement, as shown in Figure 2. At the first stage of loading, the load increment was 2 kN–5 kN and, at the second stage of loading, the load increment was 5 kN–10 kN. Electronic displacement meters were simultaneously set at the loading end and free end, respectively, with the spacing of 70 mm at the end and 100 mm in the middle.

### 2.4. Pull-Out Test Results

The failure mode and ultimate load of each specimen in the pull-out test are shown in Table 3. The specimens in Table 3 are numbered according to the concrete strength grade-reinforcement diameter-anchorage length, such as C30-12-300.

Test results of all the specimens show the tensile failure of reinforcement. The ultimate load difference of C50 and C30 indicates that, for prefabricated concrete members with larger cover thicknesses, the concrete strength grade and reinforcement diameter significantly influences the ultimate load of the bond interface between reinforcement and concrete. The ultimate load of the ribbed steel bar specimen C50-20-300 is 8.5 times higher than that of the plain steel bar specimen C50-ø20-300.

It can be estimated that the mechanical interlocking force accounts for about 88% of the bond strength between deformed steel bars and concrete. In addition, by comparing the failure modes of different specimens, it is concluded that, with the decrease in concrete strength and the increase in steel bar diameter, the failure mode of specimens gradually changes from the tensile failure of reinforcement to pull-out failure.

### 2.5. Load-Displacement Curves

**Figure 3:**
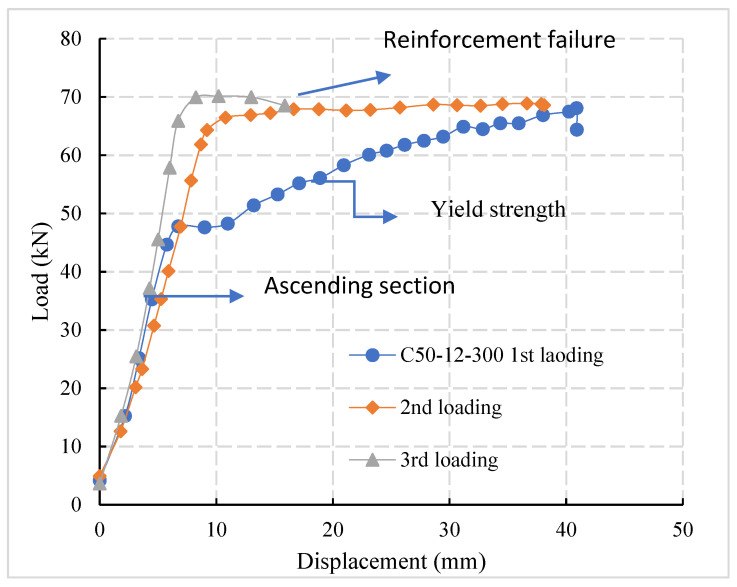
Load-displacement curve-tensile failure of reinforcement.

The load-displacement curve from the experimental results is drawn and demonstrates the tensile failure of the specimen, as shown in Figure 3. Specimen C50-12-300 is taken as an example.

Test specimens were loaded three times. At the first stage of loading, the specimen’s ductility was large and there is a yield strengthening stage before the steel bars yield. In the second stage of loading, when the load value reaches its maximum value of the first load the steel bars begin to yield and there is a longer yield deformation stage. At third stage of loading, when the load reaches the yield value the steel bars break and there is no obvious post-yield strengthening stage. The diameter of the reinforcement determines the ultimate bearing capacity of the specimen and it possesses a positive linear correlation with the cross-sectional area of the reinforcement.

### 2.6. Failure Mode

There were two main failure modes of the steel bar pull-out specimens in prefabricated concrete structures: reinforcement pull-out failure and tensile failure of the steel bar.

(1) Pull-out failure of reinforcement.

**Figure 4:**
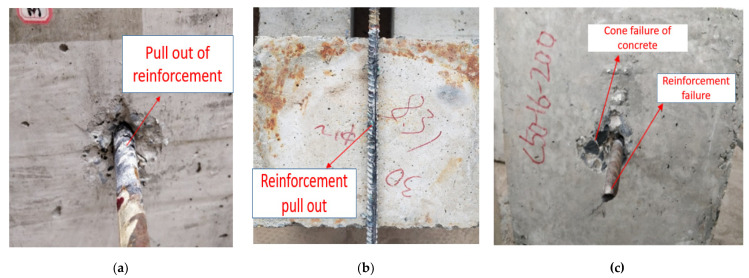
(**a**) Pull-out failure of reinforcement at loading end. (**b**) Cone-shaped failure after pulling out. (**c**) Tensile failure of reinforcement.

When the anchorage is insufficient, the pull-out force was greater than the bond interface bearing capacity of reinforced concrete and the failure of the bond interface occurs in the specimens. It can be seen from Figure 4a when the ribbed bar is pulled out, the concrete at the loading end was damaged in a cone shaped manner and there was no obvious necking of the bar. It can be seen from Figure 4b that the transverse rib of the reinforcement at the bond between the pulled-out reinforcement and concrete was intact. Under the pull-out load, the reinforcement was pulled out together with some intercostal concrete. The residual intercostal concrete accounts for about 50% of the spacing between the transverse ribs of the reinforcement.

(2) Tensile failure of the steel bar.

As shown in Figure 4c, when the reinforcement was sufficiently anchored, the bearing capacity of the reinforced concrete interface was more significant compared to the tensile bearing capacity of the reinforcement. The tensile failure of the reinforcement occurs in the specimens with a strength grade of C50.

### 2.7. Analysis of Plain and Ribbed Steel Bar Diameter

**Figure 5:**
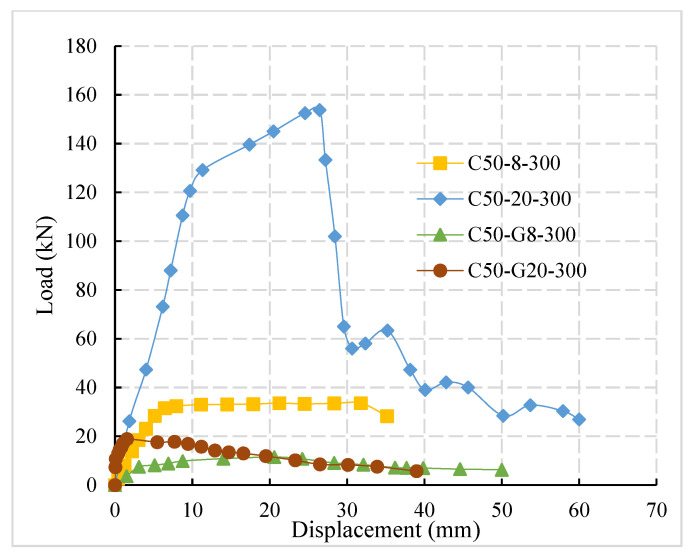
Load-displacement curve of plain and ribbed reinforcement.

Figure 5 shows the load-displacement curve of plain and ribbed reinforcement with different diameters. It can be observed from the figure that the pull-out failure of steel bars occurs in both groups of the test specimens. With the increase in steel bar diameter, the ultimate load of the specimens increases gradually. The ultimate load of C50-G8-300 and C50-G20-300 is 11.48 kN and 18.1 kN, respectively. The analysis shows that, due to the Poisson’s ratio effect of steel, the reinforcement will produce radial deformations under the pull-out force. If the diameters of steel bars were large, there would be more obvious shrinkage.

The bond interface between reinforcement-concrete was to be debonding and bond stress decreases between plain reinforcement and concrete with the increase in reinforcement diameter. Comparing the ultimate load of C50-8-300 and C50-G8-300 specimens with C50-20-300 and C50-G20-300, it is found that the interface bond strengths of plain steel bar are mainly composed of chemical bond strength and friction force between reinforced concrete, the mechanical interlocking force of bond interface is small or negligible, the bearing capacity of the bond interface between the plain steel bar and concrete is weak, and the ultimate load is far less than that of the deformed steel bar.

## 3. Modelling

### 3.1. Specimen Design

**Figure 6:**
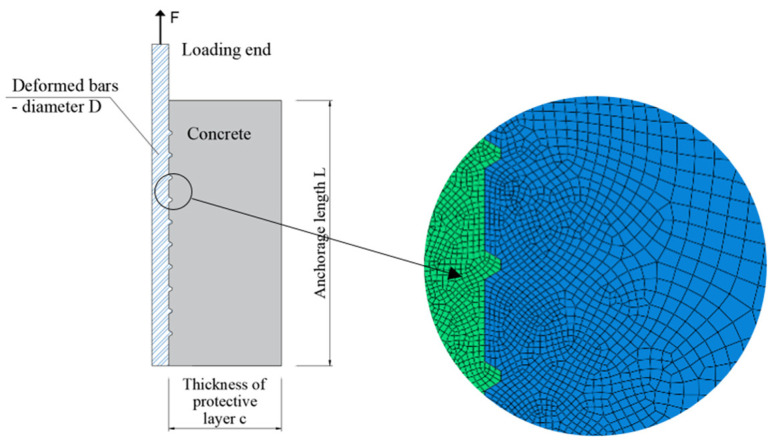
Mesh generation of the finite element model.

The finite element model (ABAQUS) of reinforced concrete pull-out specimens was established to simulate the interface bond and anchorage characteristics of steel bars within prefabricated concrete. In order to ensure the accuracy of the model and improve the calculation efficiency, the bilinear axisymmetric quadrilateral reduced integral element CGAX4R was used for both reinforcement and concrete. The mesh density of the bonding interface, reinforcement axis, and concrete edges were 0.5 mm, 0.5 mm, and 2 mm, respectively. By establishing the surface size of the transverse rib, the influences of the artificial definition of interface elements on the analysis results were reduced. The model diagram and mesh generation are shown in Figure 6. The minimum mesh size of the model was 0.4 mm.

### 3.2. Contact Problem Simulation

The detailed characteristics of the bond interface between steel and concrete were considered. The normal behavior of the contact surface was simulated by “Hard Contact” and implemented by the Classical Lagrange Multiplier Method.

The transmitted compressive stress between the contact surfaces was unlimited. When the pressure on the contact surface becomes negative or zero, the two contact surfaces were separated. The tangential behavior was simulated by “Penalty Friction.” In the Classical Coulomb Friction Model, the critical friction stress depends on the contact pressure and the elastic slip was allowed on the contact surface. Assuming that the friction coefficient μ between the contact surfaces was the same 0.1 [29], axial symmetry was adopted for boundary conditions.

### 3.3. Parameter Selection

In order to study the interface bond strength and anchorage performance between the reinforcement and the concrete of the prefabricated bridge, the larger thickness of the protective layer was adopted according to the structural characteristics. The cross-sectional dimensions of the specimens were 300 mm × 300 mm according to the prefabricated structure specification, as per experimental design. The test parameters include the diameter of reinforcement, concrete strength, and anchorage length. The concrete strength grade was C30 and C50. The reinforcement diameter was 12 mm, 16 mm, and 20 mm and the anchorage length was 150 mm, 200 mm, and 300 mm, respectively.

### 3.4. Material Constitutive Model

**Figure 7:**
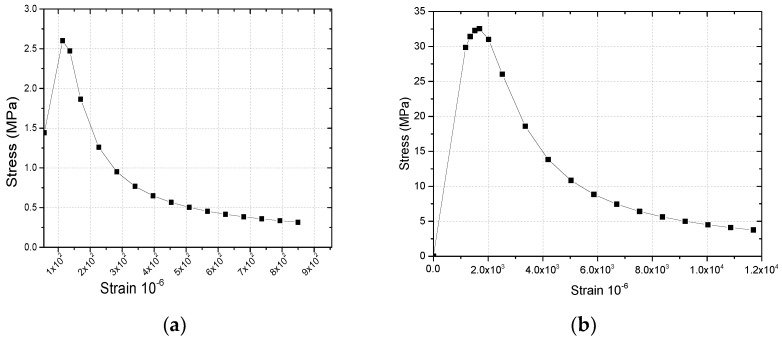
Plastic damage constitutive curve of concrete. (**a**) Compression damage constitutive curve of concrete. (**b**)Tensile damage constitutive curve of concrete.

In order to simulate the failure and crack development of the bond interface of reinforced concrete, the elastic-plastic damage constitutive model was adopted for concrete, which can be used to observe and analyze the development law of cracks by using the cloud diagram of concrete tensile damage (DAMAGET). The pull-out phenomenon of reinforced concrete’s bonding interface was observed through concrete compression damage (DAMAGEC) [30]. The constitutive model of concrete compression damage and tension damage is shown in Figure 7.

The constitutive model can be expressed in the following:

(1) $$\sigma_{t,c}=\left(1-d_{t,c}\right)E_{0}\left(\varepsilon_{t,c}-{\tilde{\varepsilon }}_{t,c}^{pl}\right)$$

where $d_{t,c}$ is the damage factor of concrete, $E_{0}$ is the elastic modulus of concrete, $\varepsilon_{t,c}$ is the concrete strain, and ${\tilde{\varepsilon }}_{t,c}^{pl}$ is the equivalent plastic strain of concrete.

In the simulation of reinforced concrete structure, the interface effect between reinforcement and concrete (such as bond-slip and locking behavior) was simulated by introducing “Tensile Hardening” into the concrete model. The tensile hardening data were defined according to the cracking strain ${\tilde{\varepsilon }}_{t}^{pl}$. The relationship between the equivalent plastic strain ${\tilde{\varepsilon }}_{t}^{ck}$ and the cracking strain ${\tilde{\varepsilon }}_{t}^{pl}$ in the model is described as follows ${\tilde{\;\varepsilon }}_{t}^{ck}$.

(2) $${\tilde{\varepsilon }}_{t}^{pl}={\tilde{\varepsilon }}_{t}^{ck}-\frac{d_{t}}{\left(1-d_{t}\right)}\frac{\sigma_{t}}{E_{0}}$$

According to the definition of compression hardening, the hardening data are defined according to the inelastic strain ${\tilde{\;\varepsilon }}_{c}^{inl}$. The relationship between the equivalent plastic strain ${\tilde{\varepsilon }}_{c}^{pl}$ and the inelastic strain ${\tilde{\varepsilon }}_{c}^{inl}$ in the model is as follows:

(3) $${\tilde{\varepsilon }}_{c}^{pl}={\tilde{\varepsilon }}_{c}^{inl}-\frac{d_{c}}{\left(1-d_{c}\right)}\frac{\sigma_{c}}{E_{0}}$$

(4) $$\sigma_{s}=\{\begin{array}{c} E_{s}\varepsilon_{s}\;\;\;\;\;\;\;\;\;\;\varepsilon_{s}\leq \;\varepsilon_{y}\;\\ f_{y}\;\;\;\;\;\;\;\;\;\;\;\;\;\;\varepsilon_{s}>\varepsilon_{y}\; \end{array}$$

where $\sigma_{s}$ is the stress of reinforcement, $E_{s}$ is the elastic modulus of reinforcement, $f_{y}$ is the yield strength of reinforcement, $\varepsilon_{s}$ is the strain of reinforcement, and $\varepsilon_{y}$ is the yield strain of reinforcement. Properties of concrete and reinforcement are indicated in Table 4.

### 3.5. Finite Element Model Validation

**Figure 8:**
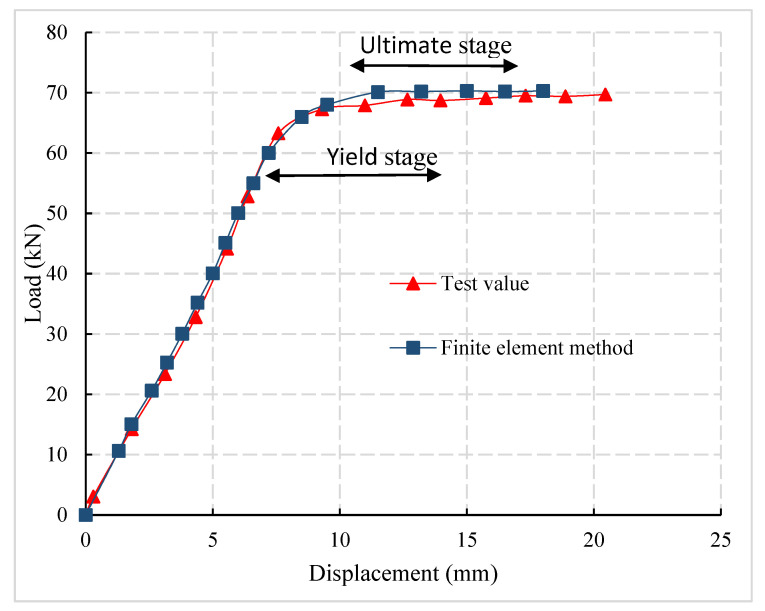
Test finite element comparison.

Taking C50-12-300 as an example, the finite element model was established. According to the results of finite element analysis, the load-displacement curve of the specimen was extracted and compared with the experimental result curve, as shown in Figure 8. It can be observed that the rising stage and ultimate load of the finite element model are consistent with that of the test specimen. The errors between the limit load of the finite element model and the experimental value were 2.4% and 0.8%, respectively.

### 3.6. Analysis of Interface Bond Failure Process

**Figure 9:**
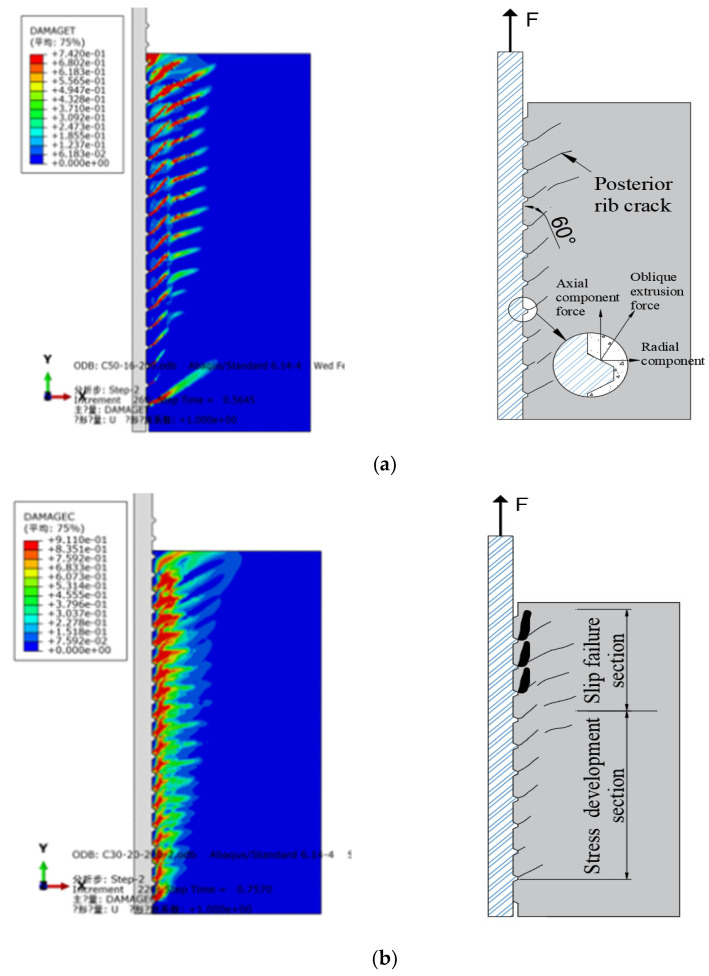
Failure process of the bonding interface. (**a**) Concrete crack development. (**b**) Interface concrete failure.

Under the pull-out load, the failure mode of the bond interface of reinforced concrete was the same as that of the experimental test results. The development of cracks cannot be directly observed in the test, but the development law of cracks can be observed and analyzed by using the cloud picture of concrete tensile damage (DAMAGET) in the finite element results. The failure appearance of the bonding interface of reinforced concrete can be observed through concrete compression damage (DAMAGEC) and the main failure mode is shown in Figure 9.

The bond strength between reinforcement and concrete was determined by the properties of the interface between them, mainly including three factors: (1) the chemical bond force between the concrete substrate and the surface coating of reinforcement; (2) the relative sliding friction resistance between reinforcement and concrete along with the interface; (3) the mechanical interlocking force caused by the unevenness of the interface between reinforcement and concrete [30,31,32,33]. The test results show that the failure modes of reinforced concrete interface bond specimens under pull-out load are mainly divided into the yield fracture of reinforcement and interface concrete failure. The bond strength of ribbed steel bar and concrete was composed of chemical bond force, friction force, and mechanical interlocking force.

At the initial loading stage, the bonding interface’s slip resistance was assumed by the chemical bonding force. In contrast, the mechanical interlocking force and friction force do not play a role temporarily. With the increase in the load, the chemical bond force fails, the bond interface slips relatively, the mechanical interlocking force and friction force begin to play a role, and the interface slip resistance is then provided by the oblique extrusion force between the transverse rib and the concrete. The axial component of the oblique extrusion force renders the concrete between the ribs subject to bending and shearing as a cantilever beam. The radial component of the oblique extrusion force results in the concrete around the reinforcement producing circumferential tensile stress. At this time, the concrete around the reinforcement was in a three-phase stress state. As shown in Figure 9a, the concrete behind the transverse rib of the steel bar was pulled by the oblique extrusion force, while the concrete in front of the rib was pressed. With the increase in the load, the radial cracks first appear behind the rib and develop along the direction of 60°, with the axial direction of the steel bar (the inclination angle of the transverse rib of the reinforcing bar). The radial crack depths of the cracks were approximately equal to the spacing between the transverse ribs of the reinforcing bar. As shown in Figure 9b, the radial failure depth was about two times the transverse rib height of the reinforcement. This failure process is called the shear bond failure of ribbed bars.

### 3.7. Load-Displacement Curve

According to the results of the finite element analysis, the load-displacement curve of the specimen was extracted, the stress characteristics and failure mechanism of different stages were analyzed, and the influences of concrete strength grade, reinforcement diameter, and anchorage length on the load-displacement curve were compared.

**Figure 10:**
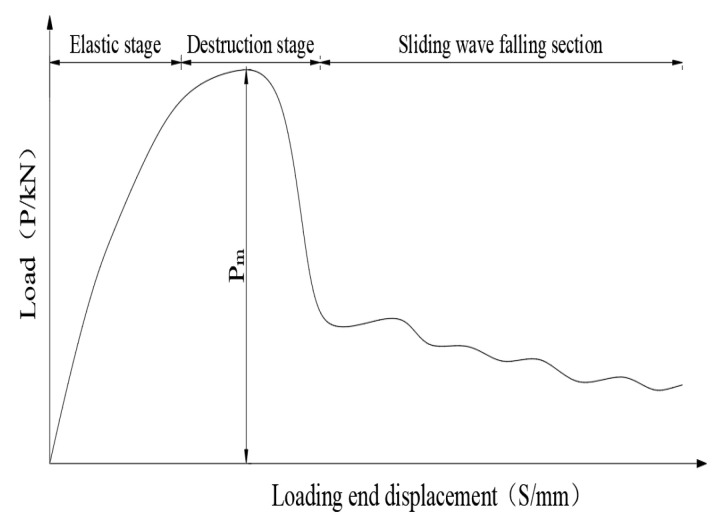
Load-displacement curve.

As shown in Figure 10, the constitutive interface model can be divided into an initial linear elastic stage and failure stage. The failure process can be divided into the micro-slip stage, internal crack slip-stage, and pull-out fluctuation decline stage. In the micro-slip stage, the slip at the loading end is minimal and there is no slip at the free end, which shows a linear phase on the load slip curve. It can be considered that the bond force gradually transfers from the near end to the far end and the adhesive force was complete during the internal crack sliding stage. When the load continues to increase, the bond force was transferred to the far end, a small amount of slip begins to appear at the far end, and the adhesive force disappears. The interface bond was mainly maintained by the friction force and mechanical interlocking forces between the concrete and reinforcement ribs. In the pull-out stage, the interface concrete was damaged, the load suddenly decreases, and the reinforcement is gradually pulled-out.

## 4. Parametric Study

### 4.1. Scope of Investigation

Parametric analysis was carried out with concrete strength, reinforcement diameter, and anchorage length as a variable. The parameters, failure mode, ultimate load, and bond strength of each specimen are shown in Table 5. The specimens in the Table are numbered according to the concrete strength grade—reinforcement diameter—anchorage length, such as C30-12-300. The calculation formula of bond stress in the Table is as follows:

(5) $$\tau =\frac{F}{\pi dl}$$

where τ is the bond strength of reinforced concrete, *F* is the pull-out load, *d* is the diameter of reinforcement, and l is the effective bond length.

### 4.2. Comparison of Load-Displacement Curves of FEM and Test Result

**Figure 11:**
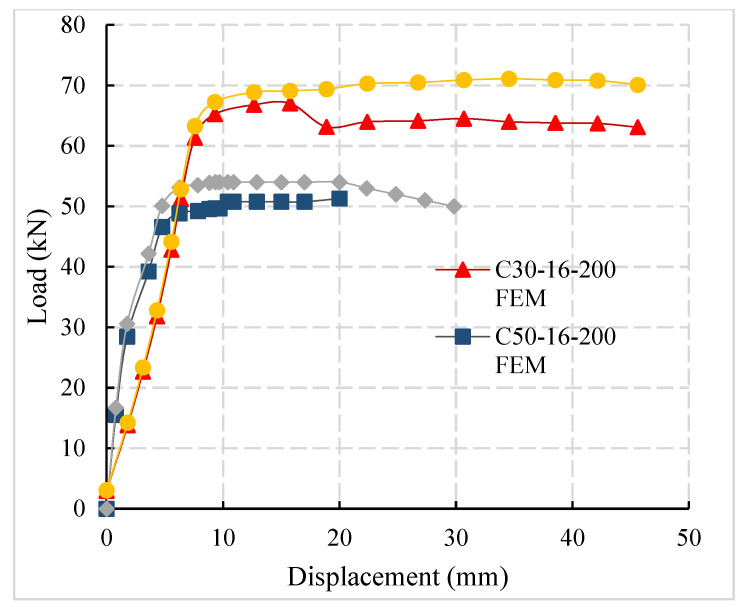
Comparison of load-displacement curves.

The load-displacement curves of the experiment and FEM are compared, which are in good agreement and shown in Figure 11. Load-displacement comparison shows that with the increase in the concrete strength, the steel bar’s pull-out failure changes into the tensile failure of the steel bar. The ultimate load-bearing capacity of the specimen increases significantly. It is concluded that in engineering practice, high-strength concrete can improve the bond strength of steel bars and reduce the requirements for anchorage length of steel bars to a certain extent.

### 4.3. Influence of the Concrete Strength Grade

**Figure 12:**
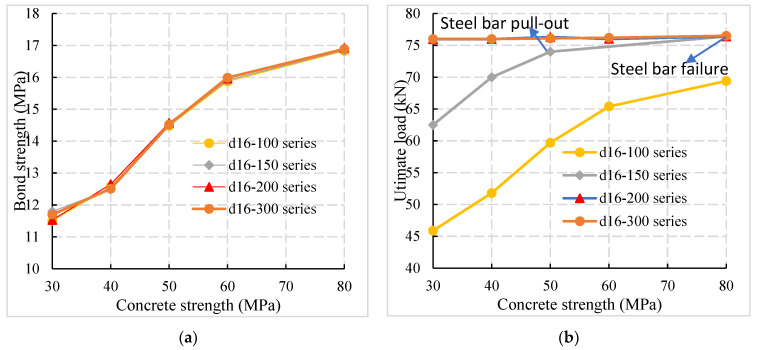
Influence of concrete strength grade. (**a**) Comparison of bond strength and concrete strength. (**b**) Ultimate load concrete strength comparison.

The bond strength and ultimate load of specimens with different concrete strengths were compared in Figure 12. The d16-100 series in Figure 12 represent five specimens with different concrete strengths with a reinforcement diameter of 16 mm and anchorage length of 100 mm.

According to Figure 12a, the bond strength between reinforcement and concrete increases with concrete strength. Compared with C30, C40, C50, and C60 specimens, the bond strength of C80 specimens increases by 44.2%, 34.7%, 16.4%, and 5.8%, respectively.

At the same time, it can be observed from Figure 12a that the correlation between the bond strength and the anchorage length of the specimens with different anchorage lengths was small for the connection reinforcement of prefabricated assembled concrete structure with larger cover thickness. The influence of anchorage length can be ignored in the calculation of bond strength. It can be observed from Figure 12b that with the increase in concrete strength, the failure mode of the specimen changes from the pull-out failure to the tensile failure of the reinforcement and the ultimate bearing capacity was significantly increased. The results show that when the pull-out failure occurs, the anchorage length was insufficient and the concrete strength possesses a significant impact on the ultimate bearing capacity of the specimens. However, when the steel bar was failing, the anchorage length was sufficient, such as the d16-200 series and d16-300 series, and the concrete strength had little influence.

### 4.4. Influence of the Reinforcement Diameter

**Figure 13:**
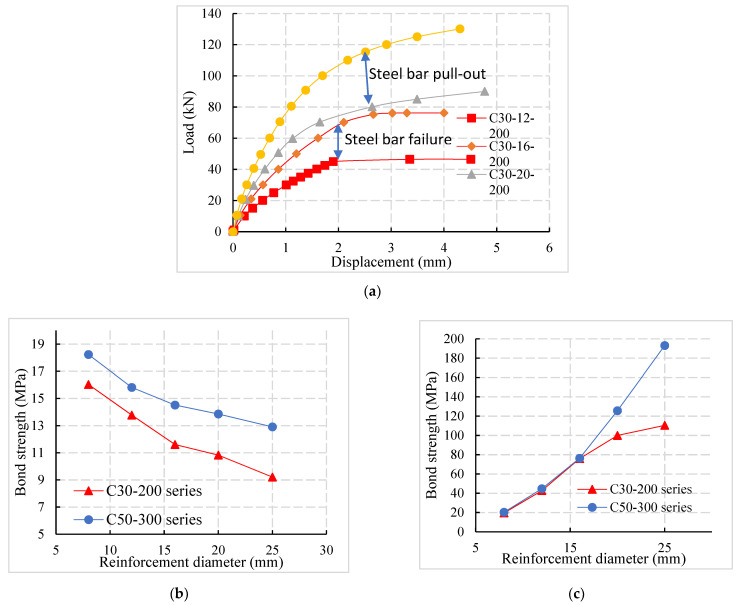
(**a**) Comparison of load-displacement curves of specimens with different reinforcement diameters. Influence of steel bar diameter: (**b**) Bond strength and steel bars diameter; (**c**) Ultimate load and steel bars diameter.

The load-displacement curves of specimens with different reinforcement diameters were compared, as shown in Figure 13a. When the anchorage length and concrete strength grades were the same, the specimens’ bearing capacity gradually increased with the increase in the reinforcement diameter. However, the failure mode of the specimens changed from the tensile failure (C30-12-200 and C30-16-200) to the pull-out failure (C30-20-200 and C30-25-200), indicating that the specimens did not pull out and the anchorage length required for failure was significantly increased; that is, the anchorage length closely related to the diameter of reinforcement.

From the previous analysis, it can be seen that the interfacial bond strength between the reinforcement and the concrete was mainly composed of the mechanical interlocking force. Shape parameters determine the mechanical interlocking force, the transverse rib’s height, and the spacing between the transverse ribs. The relative rib area (the ratio of the projected area of the transverse rib on the surface of the reinforcement to the reinforcement’s surface area) is taken as the index to evaluate the bond performance.

When the relative rib area was larger, the bond performance should be improved. According to (GB 1499.2-2018) [25], the relative rib area of reinforcement decreases with the increase in the diameter of steel bars and the bond performance between the connecting reinforcement and the concrete also decreases, as shown in Figure 13b Compared with the diameter of the specimen of 8 mm, 12 mm, 16 mm, and 20 mm, the bond strength of the 25 mm steel bar decreases by 33.9%, 28.1%, 15.6%, and 11.1%. It can be seen from Figure 13c that the ultimate load is linearly related to the cross-sectional area of the reinforcement. As the reinforcement’s diameter increases, the specimen’s failure mode changes from tensile failure to pull-out failure. The diameter of the reinforcement is no longer the main influencing factor of the ultimate load (such as the C30-200 series).

When the diameter of reinforcement increases, the bond area and mechanical interlocking depth of reinforcement concrete increases. The increment of interface bearing capacity caused by the increase in bond area was more significant than the decrease caused by the decrease in bond strength under the same conditions. It was concluded that the ultimate load still increases with the increase in reinforcement diameter.

### 4.5. Influence of Anchorage Length

**Figure 14:**
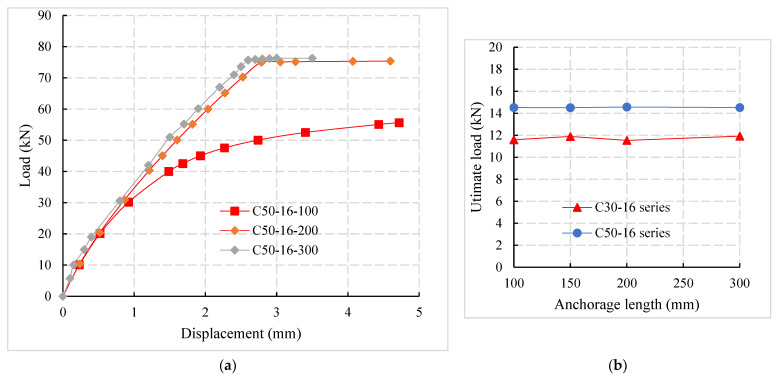

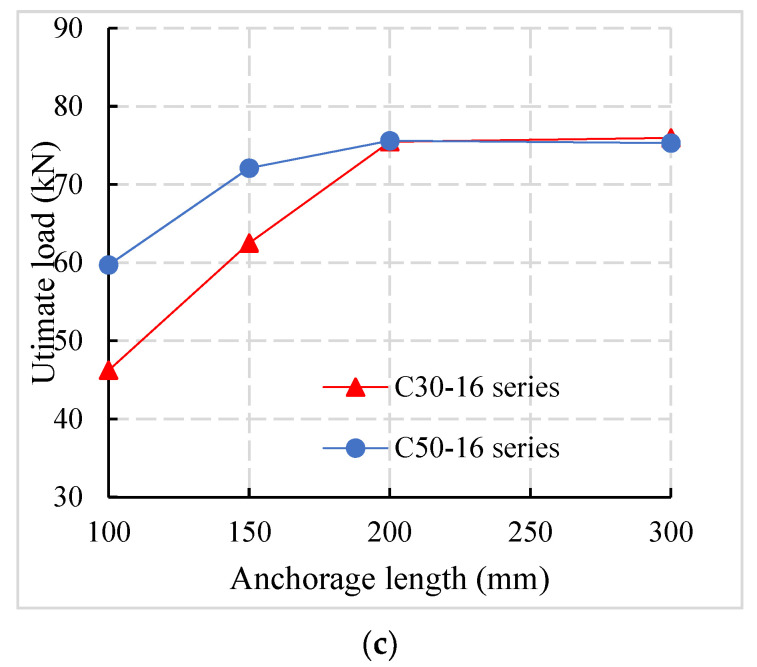
Influence of anchorage length. (**a**) Load-displacement curves for anchorage lengths. (**b**) Bond strength and anchorage length. (**c**) Relationship b/w ultimate load and anchorage length.

It can be observed from the C50-16-100 specimen in Figure 14a that when the steel bars were not sufficiently anchored, the pull-out failure of the specimen occurs. Before the steel bars were pulled out, the interface stiffness decreases rapidly with the load increase and the bond interface between reinforcement and concrete fails. When the reinforcement is fully anchored (C50-16-200 and C50-16-300 specimens in Figure 14a and the interface stiffness decreases with the increase in load before the reinforcement yields, the change amplitude was small. It can be observed from Figure 14b that the anchorage length has little effect on the bond strength between the connecting steel bar and the concrete.

By comparing the ultimate load of different specimens in Figure 14c, it was found that the ultimate load of specimens with an anchorage length of 200 mm in the C30-16 series increases by 21.5% and 64.2%, respectively, compared with specimens with anchorage lengths of 150 mm and 100 mm. It was concluded that when the anchorage length was not sufficient, the ultimate load increases with the increase in anchorage length and the failure mode of the specimen will change from pull-out failure to tensile failure.

## 5. Simplified Calculation Formula

### 5.1. Calculation Formula for the Bond Strength

For calculating the bond strength of steel bars within the concrete, scholars and codes have given the corresponding semi-empirical and semi-theoretical calculation formulas [23,24,33,34] by comprehensively considering different factors. The relevant formulas consider the concrete strength, protective layer thickness, anchorage length, and reinforcement diameter. The typical calculation formula of bond strength is shown in Table 6. The concrete structure design code [24] only calculates the bond strength between steel bar and concrete from the perspective of concrete tensile strength, without considering the influence factors such as steel bar type, steel bar diameter, anchorage length, and cover thickness. The Australian code [33] and the American code [34] considered the concrete strength, which is the ratio of concrete cover thickness to steel bar diameter, as the key index of bond strength calculation.

Although the concrete cover in the fabricated structure is sufficient, the bond strength of the connecting steel bar will not be affected due to the too-small cover thickness. Therefore, the existing formula for calculating the bond strength of reinforcement is not suitable for the calculation of the connecting reinforcement of prefabricated bridges, which will lead to the length of the reserved connecting reinforcement of prefabricated components and will have an adverse impact on the construction difficulty and assembly accuracy control.

Therefore, the existing formula of bond strength of steel bars is not suitable for calculating the connection reinforcement of prefabricated bridges, which will lead to the long reserved connection reinforcement of prefabricated members and will have an adverse effect on the construction difficulty and accuracy control of the assembly. According to the above analysis, the main factors influencing the bond strength of the prefabricated and assembled concrete structure connecting steel bars are the diameter of the reinforcement and the concrete’s strength. Therefore, the calculation formula of the bond strength of the connecting steel bar can be analyzed.

**Figure 15:**
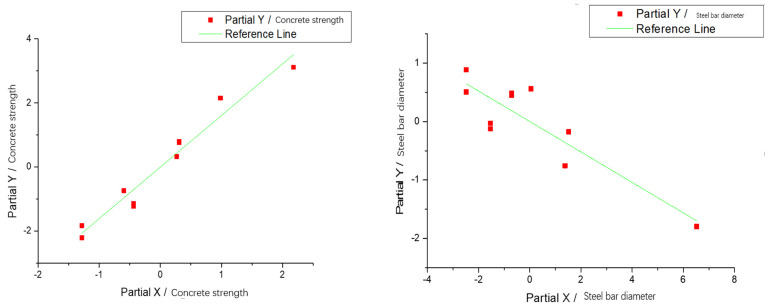
Multiple linear regression curve.

In the Table 6, $\tau_{u}$ is the interface bonding strength of reinforced concrete interface, $f_{cu}$ is the standard value of concrete compressive strength, $f_{t,r}$ is the characteristic value of concrete tensile strength, and $f_{t}$ is the splitting strength of concrete. *d* is the diameter of reinforcement and *c* is the thickness of the protective layer. The anchorage length is l and $\rho_{sv}$ is the reinforcement ratio. Multiple linear regression analysis was used to determine the influence proportion of each factor of the above formulas. The results are shown in Figure 15. It can be seen that the bond strength of the interface between reinforcement and concrete is positively correlated with the strength of concrete and negatively correlated with the diameter of reinforcement. The formula for calculating the bond strength of connecting bars in prefabricated concrete structure can be fitted as follows:

(6) $$\tau_{u}=0.1f_{cu}-0.35d+14.9$$

where $\tau_{u}$ is the bond strength of the interface between steel bar and concrete, $f_{cu}$ is the standard value of concrete compressive strength, and *d* is the diameter of the steel bar.

The minimum error between the fitting formula and the test value in literature is 2%, the maximum error is 11%, and the overall error is 7%. The minimum error is 0.6%, the maximum error is 7%, and the overall error is 4%. The average ratio of the fitting value to the test value is 1.04, and the standard deviation and coefficient of variation of the ratio are 0.06 and 5.77%, respectively. The results show that the fitting values are in good agreement with the experimental values and the dispersion is low.

**Figure 16:**
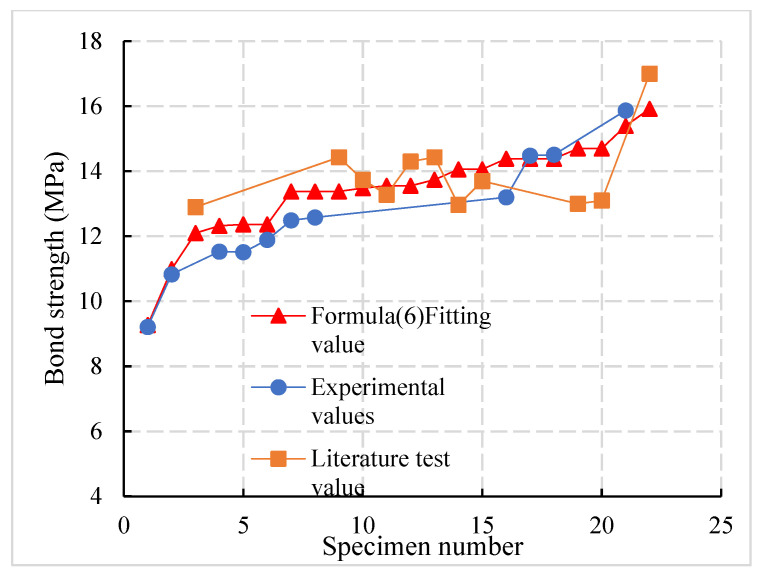
Comparison curve between fitting value and test value of bond strength.

According to the principle of the complete failure of interface, the formula (6) was compared with this paper’s test results and the references [35,36]. The comparison results are shown in Figure 16. It was found that the average ratio of the bond strength fitting value to the test value is 1.04, the standard deviation and coefficient of variation of the ratio are 0.06 and 5.77%, respectively, and the goodness of fit R^2^ between the fitting value and the measured value is 0.87, which indicates that the fitting value is in good agreement with the test value and that the dispersion is low.

**Figure 17:**
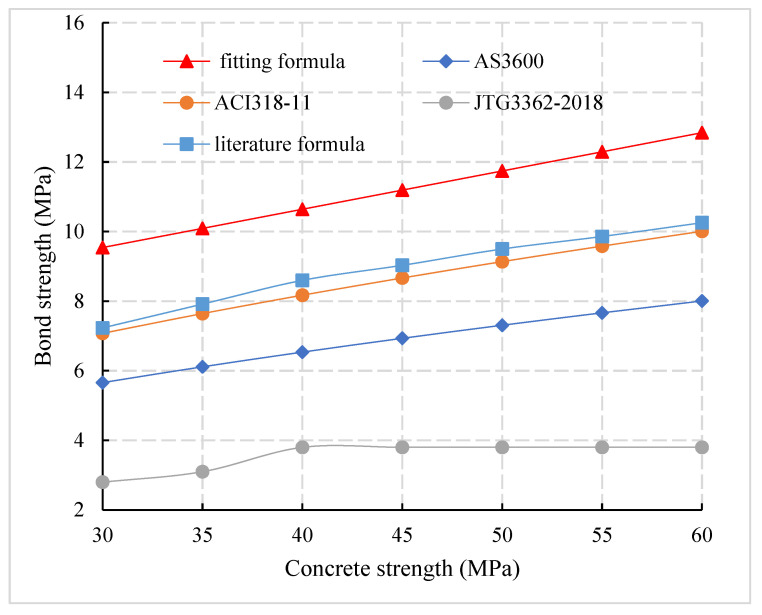
Comparison of calculation formulas of bond strength in China and abroad.

Taking the diameter of a steel bar, 25 mm, and the thickness of the protective layer 85 mm commonly used in the connection of prefabricated concrete members as an example, the differences of bond strength calculation between domestic and foreign codes and the fitting formula in this paper are shown in Figure 17. It can be observed that the bond strength calculation value of ACI 318-11 [34] is 25% and 15.9% higher than that of AS3600 [33] and JTG 3362-2018 [27], respectively, and this indicates that there are some differences in bond strength calculation between domestic and foreign codes. Compared with AS3600 [33], JTG 3362-2018 [27], and ACI318-11 [34], the bond strength of the fitting formula (6) was increased by 62.7%, 50.9%, and 30.25%, respectively, and this indicates that the calculation results of formula (6) in China and abroad are smaller without considering the influence of cover thickness, which is not suitable for the calculation of bond strength between reinforcement and concrete of prefabricated concrete members.

### 5.2. Calculation Formula for the Anchorage Length

When the bond strength between reinforcement and concrete was constant, the failure mode of the specimen was determined by the anchorage length. There was a critical length between the pull-out failure and the tensile failure of the reinforcement, which is called the critical anchorage length *l_cr_*. When the anchorage length was less than the critical anchorage length, the pull-out failure occurs.

When the anchorage length was greater than the critical anchorage length, the bearing capacity of the bonding interface was greater than the ultimate tensile load of the reinforcement and the tensile failure of the reinforcement occurs. For the calculation of the critical anchorage length of the connection reinforcement in the prefabricated concrete structure, the calculation formula of the critical anchorage length can be given according to the bond strength calculation formula as follows:

(7) $$l_{cr}=\frac{P_{u}}{\pi d\tau_{u}}=\frac{\sigma_{s}d}{4\tau_{u}}=\frac{\sigma_{s}d}{4\left(0.10f_{cu}-0.35d+14.9\right)}$$

where $P_{u}$ is the ultimate tensile load of reinforcement, $\sigma_{s}$ is the ultimate strength of reinforcement, *d* is the diameter of reinforcement, and $\tau_{u}$ is the bond strength.

The basic anchorage length *L_a_* of reinforcement specified in the code is generally determined based on the critical anchorage length calculated by the corresponding bond strength and multiplied by the corresponding safety factor. The calculation formula of the basic anchorage length of reinforcement, the ratio *L_a_*/*L_cr_* of the basic anchorage length, and the critical anchorage length in domestic and foreign codes are shown in Table 7.

The basic anchorage length of the tensile reinforcement is $l_{a}$, *n* is the concrete strength coefficient, $f_{y}$ is the design value of the tensile strength of the reinforcement, $f_{cu}$ is the standard value of the concrete compressive strength, and $f_{t}$ is the design value of the axial tensile strength of the concrete. The diameter of the anchored reinforcement is *d*, α is the shape coefficient of the anchored reinforcement, and *c* is the thickness of the protective reinforcement layer. $\psi_{t},\;\psi_{e}$, and 

**Figure 18:**
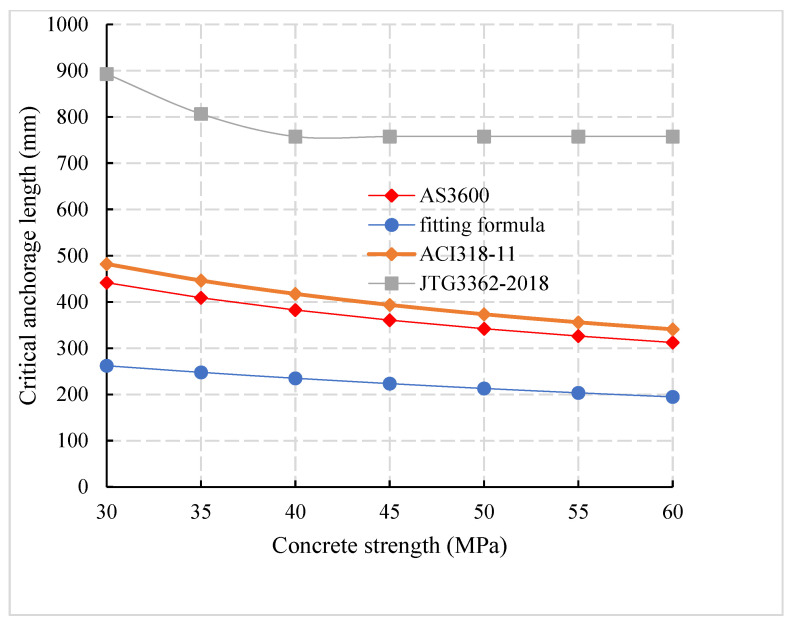
Comparison of critical anchorage length calculation between domestic and foreign codes.

$\psi_{s}$ are the reinforcement location coefficient, coating coefficient, and variety coefficient. λ is a concrete variety coefficient, $K_{tr}$ is the reinforcement coefficient, $k_{1}\;\mathrm{and}\;k_{2}$ are reinforcement location coefficient of AS3600 [33] specification, and *A* is a cross-sectional area of anchorage reinforcement. By comparison of critical anchorage length between domestic and foreign codes (Figure 18) it can be observed that the critical anchorage length corresponding to formula (7) is 0.66 times, 0.61 times, and 0.79 times of the critical anchorage length of JTG3362-2018 [27], ACI318-11 [34] and AS3600 [33] respectively, which indicates that the anchorage length requirement of the precast assembled concrete structure is far less than the standard value and special consideration should be given to it in the design and calculation.

**Figure 19:**
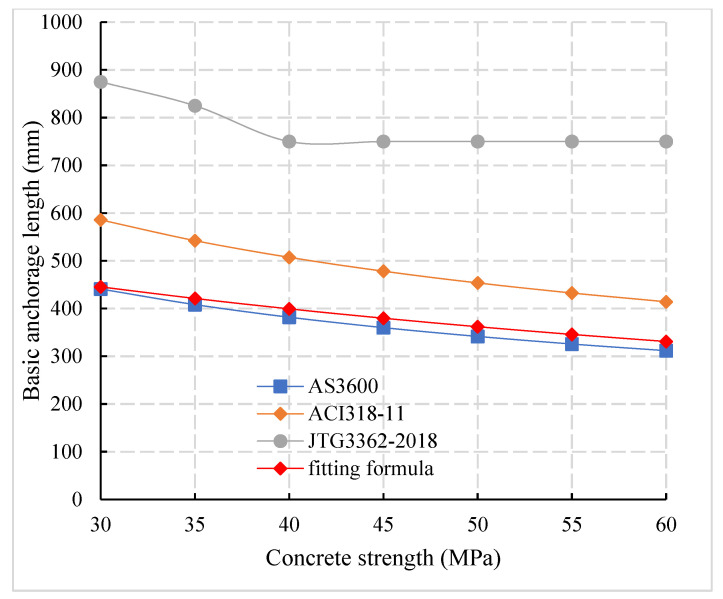
Comparison of calculation of basic anchorage length in domestic and foreign codes.

Compared with the provisions of domestic and foreign codes for the basic anchorage length of reinforcement (Figure 19), it can be observed that the basic anchorage length of JTG3362-2018 [27] is 1.61 times and 2.14 times of that of ACI318-11 [34] and AS3600 [33], respectively, compared with Figure 18 and, in Figure 18, the relationship between the critical anchorage length and the basic anchorage length in Chinese and foreign codes can be obtained. The basic anchorage length La of JTG3362-2018 [27], ACI318-11 [34], and AS3600 [33] was 1.14, 1.2, and 1.0 times of the critical anchorage length Lcr, respectively, which indicates that the Chinese code is more conservative than the foreign code. By considering 1.7 times of safety factor, the basic anchorage length of connecting reinforcement of prefabricated concrete members can be obtained. Compared with the basic anchorage length of domestic and foreign codes, the basic anchorage length of the connecting reinforcement of prefabricated bridge is 0.49 times, 0.79 times, and 1.05 times of that of JTG3362-2018 [27], ACI318-11 [34] and AS3600 [33], respectively. In Table 8, the comparison of anchorage length between domestic and foreign codes and formula (7).

In short, if the current domestic codes of prefabricated concrete will still be followed for the design of the anchorage length of prefabricated members, the size of prefabricated components will be too large, which will increase the construction cost and construction difficulty. Therefore, it is necessary to modify the anchorage length of the existing specifications. By considering the safety factor of 1.7 times, it is suggested to take it as 0.5 times the design value of the existing JTG3362-2018 [27].

## 6. Discussion

Calculation of the interface bond strength and anchorage length of steel bar within prefabricated concrete are usually considered according to the relevant provisions of the cast-in-place structure. However, the cover thickness is generally more than 50 mm larger than that of the cast-in-place structure. The complete interface failure method was used to calculate the bond strength, which may lead to the smaller calculated value of bond strength and high values of the anchorage length are not suitable for the design and construction of prefabricated bridges. Combined with experimental research and numerical analysis methods, this paper calculates the formula for interface bond strength and anchorage length by considering the main influencing factors and compares it with national and international codes. It is recommended that the basic anchorage length should be 18 d when the concrete strength grade is C35 or less and 15 d when the strength grade of the concrete is C40 or more.

## 7. Conclusions

From experimental research and numerical analysis methods, the following calculations are made.

1. It is concluded that the effect of cover thickness of the surrounding concrete is negligible for calculating interface bond strength within prefabricated structures.
2. Compared with C50-12-300 and C50-12-400, the ultimate load of C50-16-300 and C50-16-400 increased by 85.7% and 49.9%, respectively, and 19.8% and 20.8%, respectively, when compared with C30-16-300 and C30-16-400, which indicates that for precast concrete members with larger cover thicknesses, the concrete strength grade and reinforcement diameter possess a significant influence on the ultimate load of the bond interface between reinforcement and concrete.
3. The ultimate load of the ribbed steel bar specimen C50-20-300 is 8.5 times higher than that of the plain steel bar specimen C50-G20-300. It is estimated that the mechanical interlocking force accounts for about 88% of the bond strength between deformed steel bars and concrete.
4. It is concluded that domestic codes design the anchorage length of prefabricated members, leads to a large size, and increases the construction cost and construction difficulty. It is suggested to take it as 0.5 times the design value of the existing JTG3362-2018 [27] by considering the safety factor of 1.7 times.

## Figures and Tables

**Table 1:** Mix proportion of C30 and C50.

C30		Mix Ratio	C50		Mix Ratio
Cement	440	1	Cement	450	1
Sand	532	1.209	Sand	682	1.515
Aggregate	1243	2.82	Aggregate	1113	2.47
Water	185	0.420	Water	155	0.344
-	-	-	Fly ash	50	0.111

**Table 2:** Material test results of concrete and steel bar.

Specimen	Average of Elastic Modulus (MPa)	Compressive Strength (MPa)
C50	34,429	53.8
C30	32,521	31.2
Steel bar	Yield strength (MPa)	Ultimate strength (MPa)
HRB400Φ12	525	645
HRB400Φ16	605	705

**Table 3:** Failure modes of reinforced concrete pull-out specimens.

Specimen Number	Failure Mode	Ultimate Load (kN)	Specimen Number	Failure Mode	Ultimate Load (kN)
C30-12-200	Reinforcement failure	59.5	C50-12-200	Reinforcement failure	72.5
C30-12-300	Reinforcement failure	64.75	C50-12-300	Reinforcement failure	68.9
C30-16-200	Reinforcement failure	60	C50-16-200	Reinforcement failure	125.1
C30-16-300	Reinforcement failure	106.82	C50-16-300	Reinforcement failure	128
C50-ø8-300	Pull-out of a plain bar	11.48	C50-16-400	Reinforcement failure	119.7
C50-ø20-300	Pull-out of a plain bar	18.1	C50-20-300	Reinforcement failure	153.77

**Table 4:** Material properties of concrete and reinforcement.

Specimen	Poisson’s Ratio	Young’s Modulus (MPa)	Compressive Strength (MPa)
C50	0.2	34429	53.8
C30	0.2	32521	31.2
Steel bar	/	/	Ultimate strength (MPa)
HRB400Φ12	0.3	206000	645
HRB400Φ16	0.3	206000	705

**Table 5:** Analysis results.

Scheme	Failure Mode	Ultimate Load [kN]	Bond Strength /MPa	Specimen Number	Failure Mode	Ultimate Load [kN]	Bond Strength /MPa
C30-12-200	Reinforcement failure	42.71	14.78	C50-16-300	Reinforcement failure	76.3	14.52
C30-16-200	Reinforcement failure	75.96	11.53	C50-12-300	Reinforcement failure	44.71	15.82
C30-20-200	Reinforcement pull-out	99.97	10.83	C50-20-200	Reinforcement failure	124.27	13.86
C30-16-300	Reinforcement failure	74.4	11.81	C50-16-200	Reinforcement failure	75.74	14.57

**Table 6:** Calculation formula of bond strength.

Standard	Calculation Formula of Bond Strength
Australia AS3600 [33]	$\tau_{u}=0.3\times \left(0.5+c/d\right)\sqrt{f_{cu}}$
America ACI318-11 [34]	$\tau_{u}=0.08\times \left(1.2+3c/d+50d/l\right)\sqrt{f_{cu}}$
Literature [35]	$\tau_{u}=\left(1.6+0.7c/d+20\rho_{sv}\right)\left(0.82+0.9d/l\right)f_{t}$

**Table 7:** Calculation formula of basic anchorage length (domestic and foreign codes).

Standard	Calculation Formula of Basic Anchorage Length	*L_a_*/*L_cr_*
JTG3362-2018 [27]	$l_{a}=nd$	1.0
AustraliaAS3600 [33]	$l_{a}=\frac{k_{1}k_{2}f_{y}A}{(2c+d)\sqrt{f_{cu}}}\geq 25k_{1}d$	1.01
U.S.A ACI318-11 [34]	$l_{a}=\frac{\psi_{t}\psi_{e}\psi_{s}\lambda }{c+K_{tr}}\times \frac{f_{y}d^{2}}{1.1\sqrt{f_{cu}}}\geq 300\;\mathrm{mm}$	1.22

**Table 8:** Comparison of anchorage length between domestic and foreign codes.

Standard	Lcr-Formula (11)/Lcr	La/Lcr	(La-JTG)/La	1.7 Lcr(11)/La
JTG-3362-2018 [27]	0.32	1.14	1.0	0.49
ACI-318-11 [33]	0.56	1.22	1.61	0.79
AS3600 [34]	0.79	1.0	2.14	1.05

## Data Availability

Some or all data, models, or codes generated or used during the study are proprietary or confidential in nature and may only be provided with restrictions.
